# Isolation and complete genome sequence analysis of an Echovirus 29 strain isolated from a patient of Acute Flaccid Paralysis in India

**DOI:** 10.1128/mra.01110-25

**Published:** 2025-12-17

**Authors:** Madhuri S. Joshi, Rishabh Waghchaure, Pooja Umare, Abhijeet Jadhav, Alfia Fathima Ashraf, Sarah Cherian, Naveen Kumar, Babasaheb V. Tandale, Mallika Lavania

**Affiliations:** 1 ICMR – National Institute of Virology 29620, Pune, Maharashtra, India; 2 Academy of Scientific and Innovative Research, Ghaziabad, Uttar Pradesh, India; Queens College Department of Biology, Queens, New York, USA

**Keywords:** enterovirus, echovirus, outbreak, acute flaccid paralysis, India, genome analysis

## Abstract

Echovirus 29 (E29) was identified in a fecal specimen of a 15-month-old girl with acute flaccid paralysis from Akola city, Maharashtra, India. The complete genome sequence of the E29 strain, isolated using rhabdomyosarcoma cells, is being reported from India.

## ANNOUNCEMENT

Echovirus 29 (E29), a non-enveloped RNA virus of family *Picornaviridae*, is associated with illnesses ranging from asymptomatic infection to meningitis, encephalitis, acute flaccid paralysis, and respiratory disease (1). E29 has been previously detected in India during polio surveillance and is considered relevant to paralysis (2, 3). During the post-GBS outbreak investigation in Bhavanipura (Akola, Maharashtra), E29 was detected in an AFP patient’s fecal sample by qPCR and VP1 sequencing, and subsequently isolated in RD cells following WHO protocols (4). RD cells were cultured in MEM with 10% FBS and antibiotics; infected cultures showing CPE were harvested and passaged to P8. Whole-genome sequencing of the P4_E29 isolate was conducted on the Illumina MiniSeq platform (5). Viral RNA was extracted from culture supernatant using the QIAamp Viral RNA Mini Kit. After quantification, host rRNA was removed with the NEBNext depletion kit, and the RNA was purified and measured using Qubit. Libraries were prepared with the TruSeq Stranded mRNA kit (Illumina, USA) and assessed using Tapestation. Sequencing was performed on the Illumina MiniSeq (High Output Kit), and FASTQ files were analyzed with CLC Genomics Workbench v20. Raw paired-end whole-genome Illumina reads were assembled *de novo* using SPAdes genome assembler v3.15.5 (6) with default parameters, producing a single genome contig of 7,413 bp with 47.88% GC content. Genotyping using the Enterovirus Genotyping Tool identified the Indian isolate as E29.

**Fig 1:**
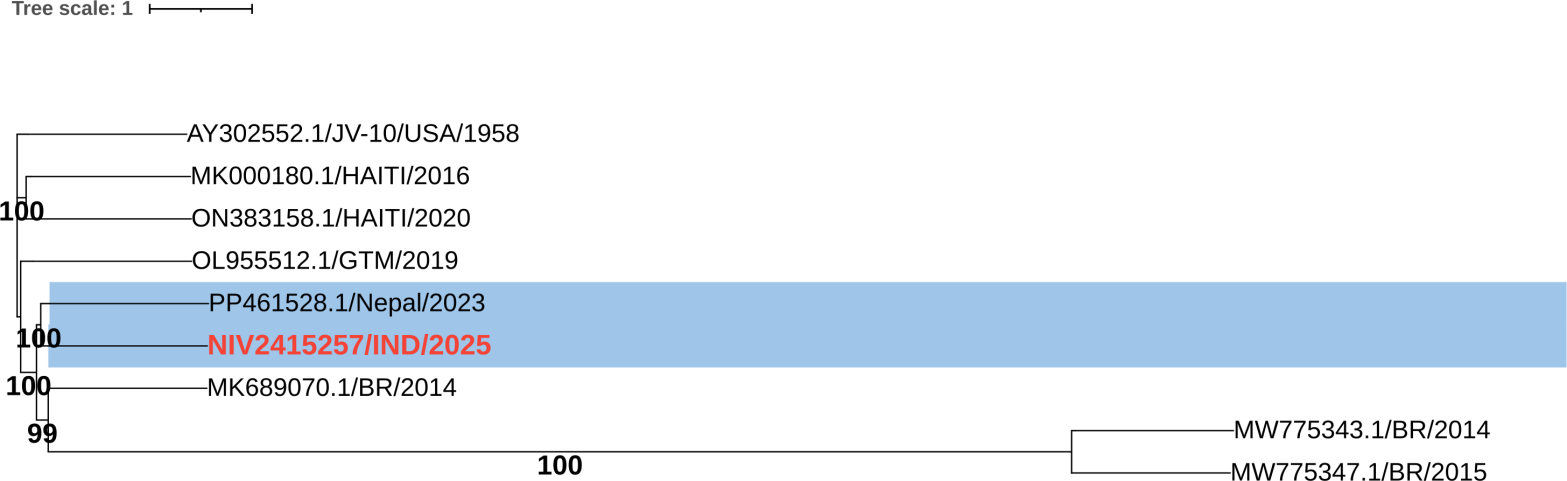
Maximum likelihood phylogenetic tree based on complete genome sequences of the E29 strain and reference sequences retrieved from the GenBank database (accession ID: prototype USA 1958 strain, AY302552.1). The sequences were aligned using MAFFT v7.5266 with default parameters. The phylogenetic tree was constructed using IQ-TREE v2.2.0 under the maximum likelihood method, with the best-fit substitution model automatically selected according to the Bayesian information criterion (BIC). Tree robustness was evaluated using 1,000 ultrafast bootstrap replicates. The final tree was visualized and annotated using Interactive Tree of Life (iTOL v6). The E29 isolate from India (NIV2415257/IND/2025) is highlighted in red font color and shows clustering with PP461528.1/Nepal/2023 strain (highlighted with light blue font color).

Although the assembled genome length (7,413 nt) matched the reference sequence, the exact 5′ and 3′ ends could not be confirmed without RACE, so it is reported as a near-complete genome. Genome annotation was performed using VAPiD (v1.6.7) with default settings and submitted to GenBank via BankIt. Sequence similarity analysis of the consensus genome using BLASTn (NCBI) revealed 99% query coverage and 86.76% nucleotide identity to Enterovirus B (E29) strain from Nepal (GenBank accession number PX230757). A maximum likelihood phylogenetic tree was generated from complete E29 genomes, aligned with MAFFT and analyzed in IQ-TREE using the BIC-selected substitution model with 1,000 ultrafast bootstraps, then visualized in iTOL. The Indian isolate (NIV2415257/IND/2025), highlighted in red, clusters closely with the Nepal 2023 strain (PP461528.1) (Fig. 1). The E29 strain clustered closely with the 2023 Nepal strain, placing it in the same well-supported lineage, along with isolates from Nepal and Brazil (2014). It showed 79% nucleotide and 96% amino acid identity with the USA 1958 prototype, indicating marked nucleotide divergence but conserved proteins. Compared with genomes from Guatemala, Haiti, Nepal, and Brazil (2014), it showed 78–82% nucleotide similarity, while sharing only ~30–32% identity with two highly divergent Brazilian strains (2014–2015) that formed a separate lineage.

A comprehensive mutation analysis at the amino acid level performed using nine whole-genome sequences available in the GenBank showed presence of 20 unique non-synonymous substitutions in the P4_E29 strain of the study. Among the 20 amino acid substitutions, 11 were in structural proteins (Table 1). The nucleotide sequence of the complete P4_E29 obtained in the study was submitted to GenBank under accession number PX230757.1.

**TABLE 1:** Unique amino acid changes in E29 strain isolated in RD cell line (NIV2415257/IND/2025)

Sample ID	Gene	Genomic coordinate	Nucleotide change	Amino acid change	AA position in region	AA position in polyprotein	HGVS notation	Type of mutation
NIV2415257/IND/2025	VP4	65	aat → agt	N → S	22	22	p.N22S	Non-synonymous
NIV2415257/IND/2025	VP2	233	tat → ttc	Y→ F	8	78	p.Y78F	Non-synonymous
NIV2415257/IND/2025	VP2	649	aat → gat	N → D	147	217	p.N217D	Non-synonymous
NIV2415257/IND/2025	VP2	676	cct → tct	P → S	156	226	p.P226S	Non-synonymous
NIV2415257/IND/2025	VP2	680	aat → acc	N → T	157	227	p.N227T	Non-synonymous
NIV2415257/IND/2025	VP2	691	gat → aat	D → N	161	231	p.D231N	Non-synonymous
NIV2415257/IND/2025	VP3	1,168	gca → caa	A → Q	58	390	p.A390Q	Non-synonymous
NIV2415257/IND/2025	VP3	1,690	gca → tca	A → S	232	564	p.A564S	Non-synonymous
NIV2415257/IND/2025	VP3	1,700	ttc → tac	F → Y	235	567	p.F567Y	Non-synonymous
NIV2415257/IND/2025	VP1	1,721	gcc → gtt	A → V	4	574	p.A574V	Non-synonymous
NIV2415257/IND/2025	VP1	2,552	ggg → gtc	G → V	281	851	p.G851V	Non-synonymous
NIV2415257/IND/2025	2A	2,656	gca → aat	A → N	24	886	p.A886N	Non-synonymous
NIV2415257/IND/2025	2A	2,717	aca → atg	T → M	44	906	p.T906M	Non-synonymous
NIV2415257/IND/2025	2B	3,107	aac → agc	N → S	24	1,036	p.N1036S	Non-synonymous
NIV2415257/IND/2025	2B	3,284	cag → cgt	Q → R	83	1,095	p.Q1095R	Non-synonymous
NIV2415257/IND/2025	2C	3,334	gga → acc	G → T	1	1,112	p.G1112T	Non-synonymous
NIV2415257/IND/2025	2C	3,462	gaa → gac	E → D	43	1,154	p.E1154D	Non-synonymous
NIV2415257/IND/2025	2C	3,959	att → acc	I → T	209	1,320	p.I1320T	Non-synonymous
NIV2415257/IND/2025	3D	5,468	agc → aac	S → N	89	1,823	p.S1823N	Non-synonymous
NIV2415257/IND/2025	3D	6,310	gtc → ata	V → I	370	2,104	p.V2104I	Non-synonymous

## Data Availability

The complete genome sequence of E29 has been submitted to GenBank via BankIt under accession number PX230757.1. The corresponding raw sequencing reads have been deposited in the Sequence Read Archive (SRA) with the BioProject ID PRJNA1348994.

## References

[1] Oyero OG, Adu FD, Ayukekbong JA. 2014. Molecular characterization of diverse species enterovirus-B types from children with acute flaccid paralysis and asymptomatic children in Nigeria. Virus Res 189:189–193. doi:10.1016/j.virusres.2014.05.029 24915283

[2] Rao CD, Yergolkar P, Shankarappa KS. 2012. Antigenic diversity of enteroviruses associated with nonpolio acute flaccid paralysis, India, 2007-2009. Emerg Infect Dis 18:1833–1840. doi:10.3201/eid1811.111457 23092622 PMC3559176

[3] Maan HS, Dhole TN, Chowdhary R. 2019. Identification and characterization of nonpolio enterovirus associated with nonpolio-acute flaccid paralysis in polio endemic state of Uttar Pradesh, Northern India. PLoS One 14:e0208902. doi:10.1371/journal.pone.0208902 30699113 PMC6353074

[4] World Health Organization. 2004. Polio laboratory manual. 4th edition. Department of Immunization, Vaccines and Biologicals Family and Community Health. https://polioeradication.org/wp-content/uploads/2017/05/Polio_Lab_Manual04.pdf.

[5] Tikute S, Deshmukh P, Chavan N, Shete A, Shinde P, Yadav P, Lavania M. 2022. Emergence of recombinant subclade D3/Y in coxsackievirus A6 strains in hand-foot-and-mouth disease (HFMD) outbreak in India, 2022. Microorganisms 12:490. doi:10.3390/microorganisms12030490 PMC10974334 38543541

[6] Bushmanova E, Antipov D, Lapidus A, Prjibelski AD. 2019. rnaSPAdes: a de novo transcriptome assembler and its application to RNA-Seq data. Gigascience 8:100. doi:10.1093/gigascience/giz100 PMC6736328 31494669

